# Tetra­aqua­(4,5-diaza­fluoren-9-one-κ^2^ *N*,*N*′)nickel(II) dinitrate

**DOI:** 10.1107/S1600536809041221

**Published:** 2009-10-17

**Authors:** Meng Guo

**Affiliations:** a Microscale Science Institute, Department of Chemistry and Chemical Engineering, Weifang University, Weifang 261061, People’s Republic of China

## Abstract

The title compound, [Ni(C_11_H_6_N_2_O)(H_2_O)_4_](NO_3_)_2_, was prepared by the reaction of Ni(NO_3_)_2_ and 4,5-diaza­fluoren-9-one (dafo). The crystal packing consists of a three-dimensional network *via* O—H⋯O hydrogen bonds between the coordin­ated water mol­ecules and the nitrate anions. The Ni atom lies on a special position (Wyckoff position 4*e*, site symmetry 2), as does the carbonyl O atom.

## Related literature

For properties of 4,5-diaza­fluoren-9-one compounds, see: Prasad *et al.* (2001 ▶, 2002 ▶). For coordination compounds with dafo, see: Prasad *et al.* (2001 ▶, 2002 ▶); Li *et al.* (2003 ▶); Wu *et al.* (2003 ▶); Zhang *et al.* (2003 ▶). For Ni—N and Ni—O bond lengths in related structures, see: Swamy *et al.* (2001 ▶); Kramer *et al.* (2002 ▶). 
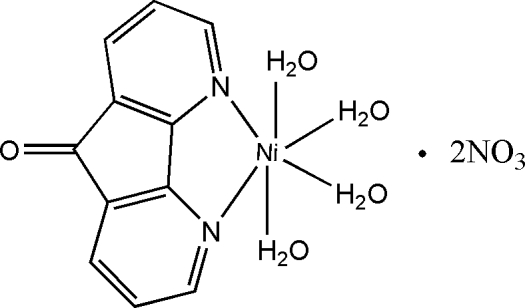


## Experimental

### 

#### Crystal data

[Ni(C_11_H_6_N_2_O)(H_2_O)_4_](NO_3_)_2_
*M* *_r_* = 436.97
Monoclinic, 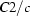

*a* = 12.904 (3) Å
*b* = 10.207 (2) Å
*c* = 13.084 (3) Å
β = 105.85 (3)°
*V* = 1657.8 (6) Å^3^
*Z* = 4
Mo *K*α radiation
μ = 1.24 mm^−1^
*T* = 293 K
0.26 × 0.25 × 0.20 mm

#### Data collection

Bruker SMART CCD area-detector diffractometer
Absorption correction: multi-scan (*SADABS*; Bruker, 1997 ▶) *T*_min_ = 0.739, *T*_max_ = 0.790
4262 measured reflections
1465 independent reflections
1303 reflections with *I* > 2σ(*I*)
*R*_int_ = 0.018

#### Refinement

*R*[*F* ^2^ > 2σ(*F* ^2^)] = 0.036
*wR*(*F* ^2^) = 0.107
*S* = 1.14
1465 reflections
124 parameters
H-atom parameters constrained
Δρ_max_ = 0.46 e Å^−3^
Δρ_min_ = −0.60 e Å^−3^

### 

Data collection: *SMART* (Bruker, 1997 ▶); cell refinement: *SAINT* (Bruker, 1997 ▶); data reduction: *SAINT*; program(s) used to solve structure: *SHELXS97* (Sheldrick, 2008 ▶); program(s) used to refine structure: *SHELXL97* (Sheldrick, 2008 ▶); molecular graphics: *SHELXTL* (Sheldrick, 2008 ▶); software used to prepare material for publication: *SHELXTL*.

## Supplementary Material

Crystal structure: contains datablocks global, I. DOI: 10.1107/S1600536809041221/fi2084sup1.cif

Structure factors: contains datablocks I. DOI: 10.1107/S1600536809041221/fi2084Isup2.hkl

Additional supplementary materials:  crystallographic information; 3D view; checkCIF report

## Figures and Tables

**Table 1:** Hydrogen-bond geometry (Å, °)

*D*—H⋯*A*	*D*—H	H⋯*A*	*D*⋯*A*	*D*—H⋯*A*
O1*W*—H2*W*1⋯O2*W*	0.85	2.40	2.838 (3)	113
O1*W*—H1*W*1⋯O1^i^	0.85	2.61	3.070 (3)	115
O1*W*—H2*W*1⋯O1^ii^	0.85	2.11	2.901 (3)	156
O2*W*—H1*W*2⋯O3^iii^	0.85	2.08	2.848 (4)	150
O2*W*—H2*W*2⋯O4^ii^	0.85	2.16	2.986 (3)	163

Symmetry codes: (i) 
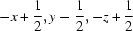
; (ii) 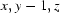; (iii) 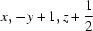.

**Fig. 1.**
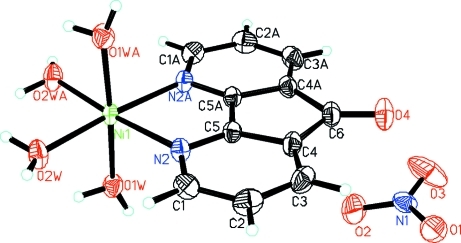
The molecular structure of the title compound with the atom-labeling scheme. Displacement ellipsoids are drawn at the 30% probability level.

**Fig. 2.**
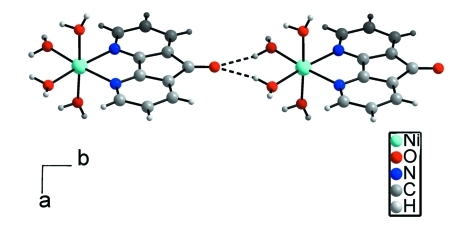
The chains formed by direct O(water)–H···O(carbonyl). H bonds as dashed lines

**Fig. 3.**
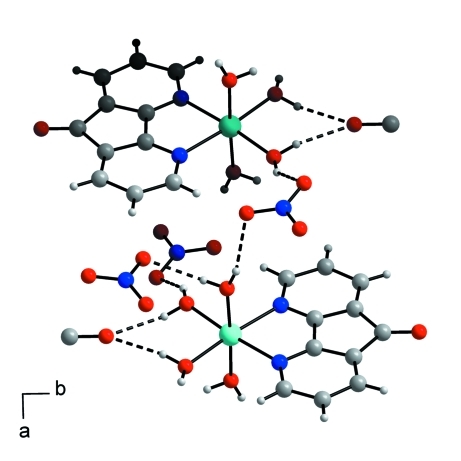
The network formed by O–H···ONO2 hydrogen bonds. H bonds as dashed lines. Only the C=O groups from the dafo ligand in the upper right and lower left corner are shown.

## supplementary crystallographic information

### Comment

4,5-diazafluoren-9-one (dafo), as well as the modified 1,10-phenanthroline (phen) neutral ligand, have allured interest in electrochemical, DNA intercalation and biological properties (Prasad *et al.*, 2002 & Prasad *et al.*, 2001). Because of the larger bite distance (2.99 Å) between two coordination nitrogen atoms, dafo provides an uncommon coordination environment, yielding a number of coordination compounds, such as two-dimensional sheets of [(dafone)_2_Cu(NCS)]_n_ (Prasad *et al.*, 2002), the zigzag chain structure of [Cu(Hnta)(afo)] (nta=nitrilotriacetate) (Li *et al.*, 2003), the helical chains of cations formed by π-π stacking interaction in [Cd(dafo)_2_(tphpo)(CH_3_COO)]ClO_4_ (tphpo=triphenylphosphine oxide) (Wu *et al.*, 2003), [Cu(dafone)_2_Cl_2_(dafoneH^+^H_2_O)_2_(ClO_4_)_2_] (Prasad *et al.*, 2001) and [Zn(C_11_H_6_N_2_O)_2_(H_2_O)_2_](NO_3_)_2_ (Zhang *et al.*, 2003). Compared to phen and 2,2^'^-bipyridine, the number of characterised metal compounds with dafo ligand is still small. Here we report the synthesis and structure of the title compound, which formes a three-dimensional network by O–H···O bonding.

The title structure (Fig. 1) features one Ni atom on a special position (Wyckoff position 4e, site symmetry 2), one 4,5-diazafluoren-9-one (dafo) ligand, four coordination water molecules and two nitrate anion. Even the dafo ligand lies on a special position (Wyckoff position 4e, site symmetry 2 with the C=O bond containing the twofold axis). Ni is coordinated by the two N atoms from dafo ligand, and four water molecules, yielding an overall tetragonally distorted octahedral geometry. The nitrogen donor atoms and two coordinated water molecules are found in the equatorial plane while the remaining two water molecules occupy the axial positions. The mean Ni—N and Ni—O bond lengths are similar to the reported (Swamy *et al.*, 2001, Kramer *et al.*, 2002). The torsion angles of O1W—Ni1—N2—C1, O2W—Ni1—N2—C1 are -91.30 (2) and -3.40 (2)°, respectively.

Hydrogen bonds are formed between the water molecules and O atoms from nitrate anions and the carbonyl group of the dafo ligand. O2w···O4 hydrogen-bond interactions link neighboring cations and form the one-dimensional chains. The anion NO_3_^-^ link these chains together by O1w···O and O2w···O hydrogen bonds, resulting in the three-dimensional network (Fig.2).

### Experimental

All commercially obtained reagent-grade chemicals were used without further purification. To a solution of dafo (0.452 g, 2.5 mmol) in methanol solution (30 ml) was added Ni(NO_3_)_2_ 6(H_2_O) (0.581 g, 2.0 mmol) in water (20 ml). The solution was stirred for 1.5 h. The resultant dark green precipitate was filtered and dried thoroughly in air. The green crystals (yield 0.79 g) were grown by slow evaporation from the water and methanol (2:3 v/v) mixed solution.

### Refinement

H atoms were positioned geometrically and allowed to ride on their parent atoms, with C—H and O—H distances of 0.93 and 0.85 Å, respectively, and with *U*_iso_(H) = 1.2*U*_eq_ of the parent atoms.

### Crystal data

**Table:**

[Ni(C_11_H_6_N_2_O)(H_2_O)_4_](NO_3_)_2_	*F*(000) = 896
*M**_r_* = 436.97	*D*_x_ = 1.751 Mg m^−^^3^
Monoclinic, *C*2/*c*	Mo *K*α radiation, λ = 0.71073 Å
Hall symbol: -C 2yc	Cell parameters from 1023 reflections
*a* = 12.904 (3) Å	θ = 2.6–25.0°
*b* = 10.207 (2) Å	µ = 1.24 mm^−^^1^
*c* = 13.084 (3) Å	*T* = 293 K
β = 105.85 (3)°	Prism, green
*V* = 1657.8 (6) Å^3^	0.26 × 0.25 × 0.20 mm
*Z* = 4	

### Data collection

**Table:**

Bruker SMART CCD area-detector diffractometer	1465 independent reflections
Radiation source: fine-focus sealed tube	1303 reflections with *I* > 2σ(*I*)
graphite	*R*_int_ = 0.018
φ and ω scans	θ_max_ = 25.0°, θ_min_ = 2.6°
Absorption correction: multi-scan (*SADABS*; Bruker, 1997)	*h* = −15→14
*T*_min_ = 0.739, *T*_max_ = 0.790	*k* = −12→6
4262 measured reflections	*l* = −15→15

### Refinement

**Table:**

Refinement on *F*^2^	Primary atom site location: structure-invariant direct methods
Least-squares matrix: full	Secondary atom site location: difference Fourier map
*R*[*F*^2^ > 2σ(*F*^2^)] = 0.036	Hydrogen site location: inferred from neighbouring sites
*wR*(*F*^2^) = 0.107	H-atom parameters constrained
*S* = 1.14	*w* = 1/[σ^2^(*F*_o_^2^) + (0.0607*P*)^2^ + 2.0108*P*] where *P* = (*F*_o_^2^ + 2*F*_c_^2^)/3
1465 reflections	(Δ/σ)_max_ < 0.001
124 parameters	Δρ_max_ = 0.46 e Å^−^^3^
0 restraints	Δρ_min_ = −0.60 e Å^−^^3^

### Special details

**Table:**

Geometry. All e.s.d.'s (except the e.s.d. in the dihedral angle between two l.s. planes) are estimated using the full covariance matrix. The cell e.s.d.'s are taken into account individually in the estimation of e.s.d.'s in distances, angles and torsion angles; correlations between e.s.d.'s in cell parameters are only used when they are defined by crystal symmetry. An approximate (isotropic) treatment of cell e.s.d.'s is used for estimating e.s.d.'s involving l.s. planes.
Refinement. Refinement of *F*^2^ against ALL reflections. The weighted *R*-factor *wR* and goodness of fit *S* are based on *F*^2^, conventional *R*-factors *R* are based on *F*, with *F* set to zero for negative *F*^2^. The threshold expression of *F*^2^ > σ(*F*^2^) is used only for calculating *R*-factors(gt) *etc*. and is not relevant to the choice of reflections for refinement. *R*-factors based on *F*^2^ are statistically about twice as large as those based on *F*, and *R*- factors based on ALL data will be even larger.

### Fractional atomic coordinates and isotropic or equivalent isotropic displacement parameters (Å^2^)

**Table:**

	*x*	*y*	*z*	*U*_iso_*/*U*_eq_	
Ni1	0.0000	0.25222 (4)	0.2500	0.0324 (2)	
O4	0.0000	0.8551 (3)	0.2500	0.0626 (9)	
N2	0.06304 (18)	0.4111 (2)	0.35714 (17)	0.0364 (5)	
C1	0.1215 (2)	0.4279 (3)	0.4587 (2)	0.0457 (7)	
H1A	0.1466	0.3544	0.5000	0.055*	
C2	0.1455 (3)	0.5512 (3)	0.5037 (2)	0.0532 (8)	
H2A	0.1856	0.5581	0.5742	0.064*	
C3	0.1109 (2)	0.6640 (3)	0.4459 (2)	0.0509 (7)	
H3A	0.1265	0.7467	0.4759	0.061*	
C4	0.0525 (2)	0.6478 (3)	0.3418 (2)	0.0398 (6)	
C5	0.0318 (2)	0.5207 (2)	0.3050 (2)	0.0338 (6)	
C6	0.0000	0.7384 (4)	0.2500	0.0452 (10)	
O1W	0.13445 (18)	0.24757 (16)	0.19659 (18)	0.0457 (5)	
H1W1	0.1848	0.2789	0.1735	0.055*	
H2W1	0.1543	0.1723	0.2223	0.055*	
O2W	0.07053 (17)	0.11039 (19)	0.35806 (16)	0.0494 (5)	
H1W2	0.1031	0.0988	0.4233	0.059*	
H2W2	0.0398	0.0387	0.3341	0.059*	
N1	0.1702 (2)	0.9279 (3)	0.1387 (2)	0.0557 (7)	
O1	0.2159 (2)	0.9812 (3)	0.2222 (2)	0.0748 (8)	
O2	0.1842 (2)	0.8094 (3)	0.1304 (3)	0.0966 (11)	
O3	0.1130 (3)	0.9928 (4)	0.0676 (2)	0.1121 (13)	

### Atomic displacement parameters (Å^2^)

**Table:**

	*U*^11^	*U*^22^	*U*^33^	*U*^12^	*U*^13^	*U*^23^
Ni1	0.0441 (3)	0.0220 (3)	0.0305 (3)	0.000	0.0092 (2)	0.000
O4	0.093 (2)	0.0298 (16)	0.073 (2)	0.000	0.0366 (19)	0.000
N2	0.0461 (12)	0.0296 (11)	0.0330 (11)	0.0009 (9)	0.0098 (9)	0.0010 (9)
C1	0.0495 (15)	0.0509 (17)	0.0345 (14)	0.0049 (13)	0.0078 (11)	0.0055 (12)
C2	0.0532 (17)	0.067 (2)	0.0363 (15)	−0.0054 (15)	0.0063 (13)	−0.0120 (15)
C3	0.0580 (17)	0.0455 (17)	0.0504 (17)	−0.0118 (14)	0.0172 (14)	−0.0173 (14)
C4	0.0476 (14)	0.0297 (13)	0.0458 (15)	−0.0041 (11)	0.0190 (12)	−0.0067 (11)
C5	0.0395 (13)	0.0297 (13)	0.0336 (13)	−0.0008 (10)	0.0123 (11)	−0.0028 (10)
C6	0.055 (2)	0.029 (2)	0.060 (3)	0.000	0.030 (2)	0.000
O1W	0.0526 (12)	0.0386 (12)	0.0502 (13)	−0.0020 (8)	0.0214 (10)	0.0063 (8)
O2W	0.0717 (14)	0.0324 (10)	0.0403 (11)	0.0043 (9)	0.0089 (9)	0.0060 (9)
N1	0.0442 (13)	0.0670 (18)	0.0534 (16)	0.0116 (13)	0.0092 (12)	−0.0186 (14)
O1	0.0706 (15)	0.0778 (18)	0.0633 (15)	0.0083 (13)	−0.0032 (12)	−0.0237 (13)
O2	0.090 (2)	0.070 (2)	0.134 (3)	−0.0015 (16)	0.0374 (19)	−0.052 (2)
O3	0.103 (2)	0.172 (3)	0.0543 (16)	0.077 (2)	0.0078 (15)	0.0039 (19)

### Geometric parameters (Å, °)

**Table:**

Ni1—O1W^i^	2.040 (2)	C3—C4	1.374 (4)
Ni1—O1W	2.040 (2)	C3—H3A	0.9300
Ni1—O2W^i^	2.054 (2)	C4—C5	1.383 (3)
Ni1—O2W	2.054 (2)	C4—C6	1.521 (4)
Ni1—O2W	2.054 (2)	C5—C5^i^	1.450 (5)
Ni1—N2	2.153 (2)	C6—C4^i^	1.521 (4)
Ni1—N2^i^	2.153 (2)	O1W—H1W1	0.8502
O4—C6	1.191 (4)	O1W—H2W1	0.8501
N2—C5	1.315 (3)	O2W—O2W	0.000 (4)
N2—C1	1.347 (4)	O2W—H1W2	0.8501
C1—C2	1.388 (4)	O2W—H2W2	0.8500
C1—H1A	0.9300	N1—O3	1.214 (4)
C2—C3	1.382 (5)	N1—O1	1.219 (4)
C2—H2A	0.9300	N1—O2	1.231 (4)
			
O1W^i^—Ni1—O1W	177.33 (10)	C3—C2—C1	121.5 (3)
O1W^i^—Ni1—O2W^i^	87.77 (8)	C3—C2—H2A	119.3
O1W—Ni1—O2W^i^	90.35 (9)	C1—C2—H2A	119.3
O1W^i^—Ni1—O2W	90.35 (9)	C4—C3—C2	116.7 (3)
O1W—Ni1—O2W	87.77 (8)	C4—C3—H3A	121.7
O2W^i^—Ni1—O2W	90.39 (12)	C2—C3—H3A	121.7
O1W^i^—Ni1—O2W	90.35 (9)	C3—C4—C5	117.3 (3)
O1W—Ni1—O2W	87.77 (8)	C3—C4—C6	135.6 (3)
O2W^i^—Ni1—O2W	90.39 (12)	C5—C4—C6	107.1 (2)
O2W—Ni1—O2W	0.00 (12)	N2—C5—C4	127.9 (2)
O1W^i^—Ni1—N2	89.99 (8)	N2—C5—C5^i^	121.67 (14)
O1W—Ni1—N2	92.01 (8)	C4—C5—C5^i^	110.40 (16)
O2W^i^—Ni1—N2	175.31 (8)	O4—C6—C4^i^	127.46 (15)
O2W—Ni1—N2	93.74 (9)	O4—C6—C4	127.46 (15)
O2W—Ni1—N2	93.74 (9)	C4^i^—C6—C4	105.1 (3)
O1W^i^—Ni1—N2^i^	92.01 (8)	Ni1—O1W—H1W1	156.5
O1W—Ni1—N2^i^	89.99 (8)	Ni1—O1W—H2W1	94.5
O2W^i^—Ni1—N2^i^	93.74 (9)	H1W1—O1W—H2W1	107.7
O2W—Ni1—N2^i^	175.31 (8)	O2W—O2W—Ni1	0(10)
O2W—Ni1—N2^i^	175.31 (8)	O2W—O2W—H1W2	0.0
N2—Ni1—N2^i^	82.22 (12)	Ni1—O2W—H1W2	142.5
C5—N2—C1	114.4 (2)	O2W—O2W—H2W2	0.0
C5—N2—Ni1	107.21 (16)	Ni1—O2W—H2W2	106.1
C1—N2—Ni1	138.41 (19)	H1W2—O2W—H2W2	107.7
N2—C1—C2	122.2 (3)	O3—N1—O1	119.1 (3)
N2—C1—H1A	118.9	O3—N1—O2	122.8 (3)
C2—C1—H1A	118.9	O1—N1—O2	118.1 (3)

Symmetry codes: (i) −*x*, *y*, −*z*+1/2.

### Hydrogen-bond geometry (Å, °)

**Table:**

*D*—H···*A*	*D*—H	H···*A*	*D*···*A*	*D*—H···*A*
O1W—H2W1···O2W	0.85	2.40	2.838 (3)	113
O1W—H1W1···O1^ii^	0.85	2.61	3.070 (3)	115
O1W—H2W1···O1^iii^	0.85	2.11	2.901 (3)	156
O2W—H1W2···O3^iv^	0.85	2.08	2.848 (4)	150
O2W—H2W2···O4^iii^	0.85	2.16	2.986 (3)	163

Symmetry codes:  (ii) −*x*+1/2, *y*−1/2, −*z*+1/2; (iii) *x*, *y*−1, *z*; (iv) *x*, −*y*+1, *z*+1/2.
